# Supplementary material for: Age-Dependent Decrease in Hepatic Geranylgeranoic Acid Content in C3H/HeN Mice and Its Oral Supplementation Prevents Spontaneous Hepatoma
Source: Metabolites. 2021 Sep 17;11(9):634. doi: 10.3390/metabo11090634 (PMC8471303; doi:10.3390/metabo11090634)
Supplement: Supplementary file 1 [file metabolites-11-00634-s001.zip › metabolites-1365420-supplementary.pdf]

Supplementary Material: Age-Dependent Decrease in Hepatic Geranylgeranoic Acid Content in C3H/HeN Mice and its Oral Supplementation Pre-vents Spontaneous Hepatoma

Yuki Tabata, Masahide Omori and Yoshihiro Shidoji

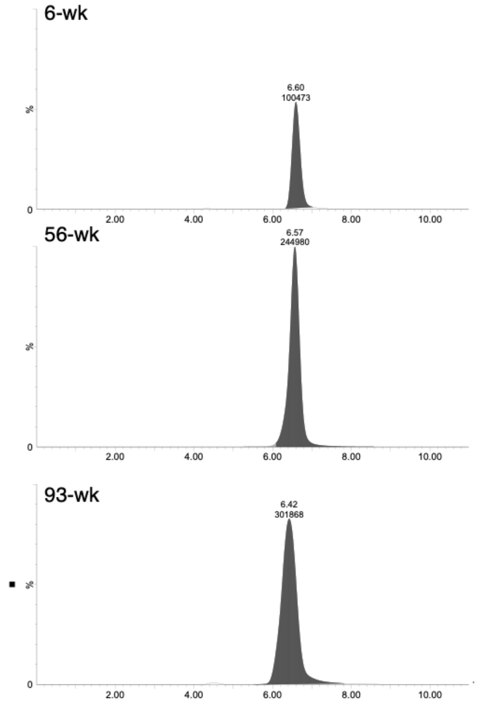

Figure S1. Representative LC/MS/MS chromatograms of the 6-, 56- and 93-week old mouse hepatic lipid extracts tracing arachidonic acid (m/z: 303-259). The chromatograms show a peak height of 7.87 x 10^5 as 100%.

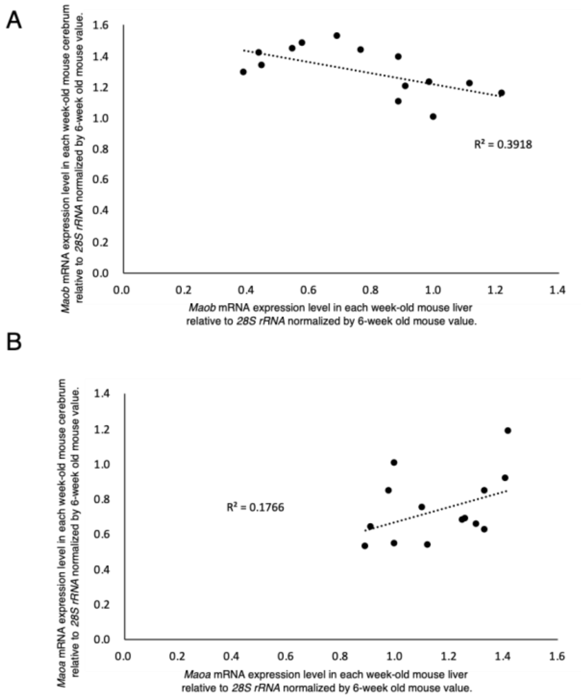

Figure S2. A: Association of hepatic *Maob* mRNA with cereberum *Maob* mRNA levels. B: Association of hepatic *Maoa* mRNA with cerebrum *Maoa* mRNA levels. Each point represent the average value of each age group (n = 5). *Maoa*, monoamine oxidase a; *Maob*, monoamine oxidase b.

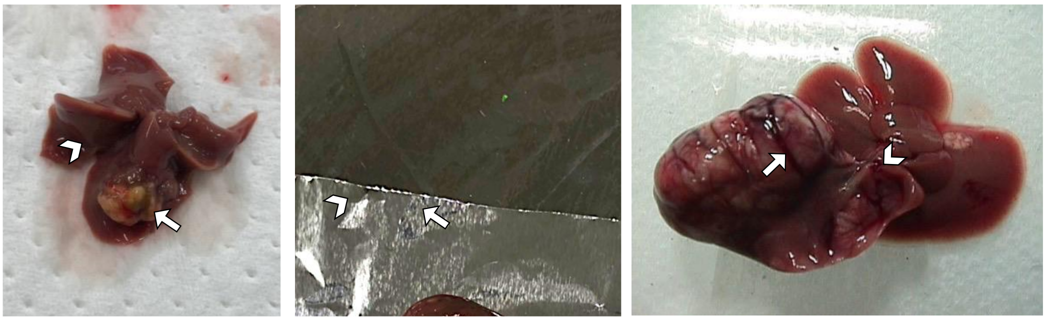

Figure S3. Representative normal tissue (arrowhead) and tumor tissue (arrow) of male C3H/HeN mice used for measurement of endogenous GGA and *Maoa/Maob* mRNA levels.

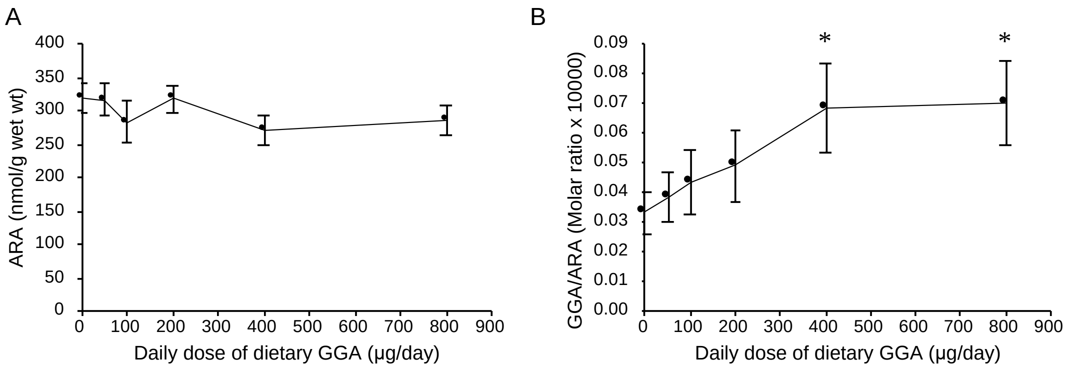

Figure S4. Hepatic free ARA levels (A) and GGA/ARA molar ratio (B) in mice liver are plotted along with daily dose of orally administered GGA. The results are expressed as the mean ± SD (n = 3). *P < 0.05 versus mice fed a control diet (ANOVA with post hoc Scheffe). ARA, arachidonic acid; GGA, geranylgeranoic acid;

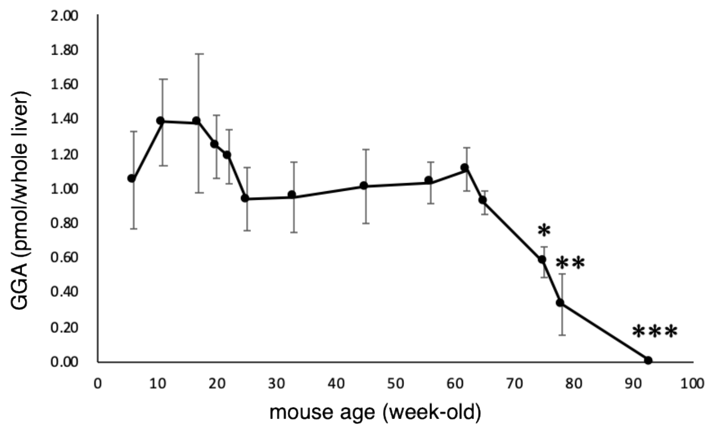

Figure S5. Age-dependent changes of hepatic GGA content per C3H/HeN mouse. Endogenous GGA contents in the whole liver of each week-old male C3H/HeN mouse are plotted. All points represent the mean ± SEM (n = 5). *P < 0.05, **P < 0.01 and ***P < 0.001 versus 6-week old mice (ANOVA with post hoc Scheffe). GGA, geranylgeranoic acid.

Supplementary Table S1. The nucleotide sequences of each primer used for RT-qPCR.

Genes	Primer	Sequence (5' – 3')
Maoa	F	GGTCCTCCTTGGGGATAAAG
	R	TTCTCTCAGGTGGAAGCTCTG
Maob	F	GCACTGAAACAGCCTCACAC
	R	TCGTGCAGGGACATCCAAAG
28S rRNA	F	GCTCAGTACGAGAGGAACCG
	R	AGAGGCGTTCAGTCATAATC

F: forward primer, R: reverse primer

Maoa, monoamine oxidase a; *Maob*, monoamine oxidase b.

Supplementary Table S2. The condition of thermal cycler for RT-qPCR of *Maoa*, *Maob and 28s rRNA*.

	Temperature, Duration	Slope
Denature	95ºC, 600 s	20ºC / s
PCR (40 cycles)	95ºC, 15 s	20ºC / s
	65ºC, 60 s	20ºC / s
Melting	95ºC, 0 s	20ºC / s
	57ºC, 15 s	20ºC / s
	98ºC, 0 s	-
Cooling	40ºC, 30 s	20ºC / s

Maoa, monoamine oxidase a; *Maob*, monoamine oxidase b.
